# A Wireless Intracranial Brain Deformation Sensing System for Blast-Induced Traumatic Brain Injury

**DOI:** 10.1038/srep16959

**Published:** 2015-11-20

**Authors:** S. Song, N. S. Race, A. Kim, T. Zhang, R. Shi, B. Ziaie

**Affiliations:** 1 School of Electrical and Computer Engineering, Purdue University, West Lafayette, IN, USA; 2 Birck Nanotechnology Center, West Lafayette, IN, USA; 3 Weldon School of Biomedical Engineering, Purdue University, West Lafayette, IN, USA; 4 Department of Basic Medical Sciences, Purdue University, West Lafayette, IN, USA; 5 School of Medicine, Indiana University, Indianapolis, IN, USA

## Abstract

Blast-induced traumatic brain injury (bTBI) has been linked to a multitude of delayed-onset neurodegenerative and neuropsychiatric disorders, but complete understanding of their pathogenesis remains elusive. To develop mechanistic relationships between bTBI and post-blast neurological sequelae, it is imperative to characterize the initiating traumatic mechanical events leading to eventual alterations of cell, tissue, and organ structure and function. This paper presents a wireless sensing system capable of monitoring the intracranial brain deformation in real-time during the event of a bTBI. The system consists of an implantable soft magnet and an external head-mounted magnetic sensor that is able to measure the field in three dimensions. The change in the relative position of the soft magnet WITH respect to the external sensor as the result of the blast wave induces changes in the magnetic field. The magnetic field data in turn is used to extract the temporal and spatial motion of the brain under the blast wave in real-time. The system has temporal and spatial resolutions of 5 μs and 10 μm. Following the characterization and validation of the sensor system, we measured brain deformations in a live rodent during a bTBI.

With the continual advancement and lowering cost of small explosives, particularly improvised explosive devices, blast-induced traumatic brain injury (bTBI), already one of the most significant wounds throughout Operation Enduring Freedom (OEF) and Operation Iraqi Freedom (OIF), has become increasingly prevalent. Approximately 167,000 bTBI cases have been documented during OEF and OIF deployments alone^1^,^2^, and the true number of bTBI incidents is expected to be even higher due to under-reporting and the fact that they can also occur during training exercises^3^. Post-bTBI consequences are dire, ranging from neurodegenerative diseases such as chronic traumatic encephalopathy to neuropsychiatric alterations such as depression, anxiety, and more^4^,^5^. These risks pose a substantial public health burden upon military members’ return to civilian life, as the conditions are generally chronic and involve lengthy and costly treatment courses both in terms of dollars and quality of life. To pursue targeted innovation of new preventative, diagnostic, and therapeutic measures, we must first develop greater understanding of bTBI pathogenesis, its initiating mechanical events, and the links between blast-induced damage and subsequent neuropathologies.

Blast waves’ ability to cause primary injury to brain has been debated without a consensus in terms of whether or not the blast wave can propagate through the skull, or if the injury can be prevented or mitigated by a neck fixation^6^. Although it has been demonstrated that exposures to blast waves compromise the blood-brain barrier and cause both dynamic short-term and sustained long-term intracranial pressure rises hypothesized to lead to axonal injuries, knowledge regarding the pathobiological mechanisms of primary bTBI is limited^7^,^8^,^9^. This can largely be attributed to the experimental challenges of studying the brain deformation caused by blast waves in real time due to the presence of the skull, and high temporal and spatial resolution requirements^6^,^7^,^10^,^11^,^12^. Such requirements render traditional imaging methods inapplicable to study brain dynamics in the primary bTBI. Alternatives are simulations or controlled experimental models to mimic the actual injury. Simulation models to date have suffered from a lack of experimental data to validate the results^7^,^13^ as few experimental bTBI biomechanics investigations have been conducted in animal models.

In this paper, we present a sensor system capable of real-time *in vivo* measurements of the intracranial brain deformation using an implantable elastomeric polymeric magnet and three external 3-axis giant magnetoresistance sensor (GMR). The sensor system was characterized, validated, and applied to three different experimental models (3D-printed skull with agarose gel, dead rats, and live rats) to measure intracranial brain deformations during blast waves generated via an open-ended shock tube system.

## Results

**Figure 1:**
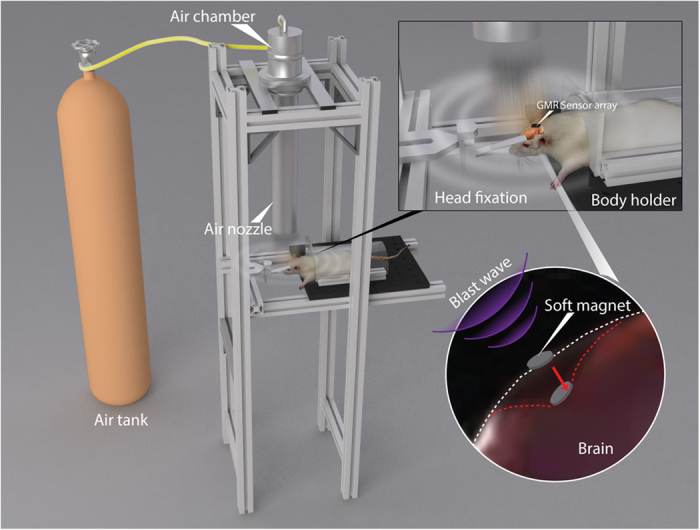
Schematic of intracranial brain deformation sensor system and blast model. The soft magnet is implanted on the dura mater and the GMR array is fixed on the skull. The change in the relative position due to the brain deformation under a blast wave can be measured by the change in magnetic field.

To achieve the systematic study of brain deformation of rat models under bTBI, the intracranial deformation sensor system is integrated into a validated custom-built blast wave generator as shown in Fig. 1^14^. The blast wave is generated by flowing pressurized air into the air chamber from the air tank. When the plastic membrane separating the air chamber and nozzle ruptures, a blast wave is delivered through the air nozzle. The test arrangement, rats, are immobilized under the nozzle by a head fixation and a body holder^14^. The stereotaxic head fixation isolates primary shock wave-induced deformation from blast wind acceleration-induced brain motion^14^. The first component of the sensor system, GMR sensor array, is fixed on the skull (Fig. 1: top-right). The second component, the soft magnet, sits on top of the dura mater and follows the brain deformation during blast exposure (Fig. 1: bottom-right). The brain deformation is not expected to be a traditional rigid transformation but rather viscoelastic and differ from location to location in acceleration, velocity, and/or direction. To measure these heterogeneous deformations, our system reports the local brain deformation at the site of implantation by tracking the soft magnet displacement. The displacement of the soft magnet results in a change in the magnetic field at the location of the GMR array with the corresponding position obtained in-real time allowing the measurement of brain deformation during bTBI.

**Figure 2:**
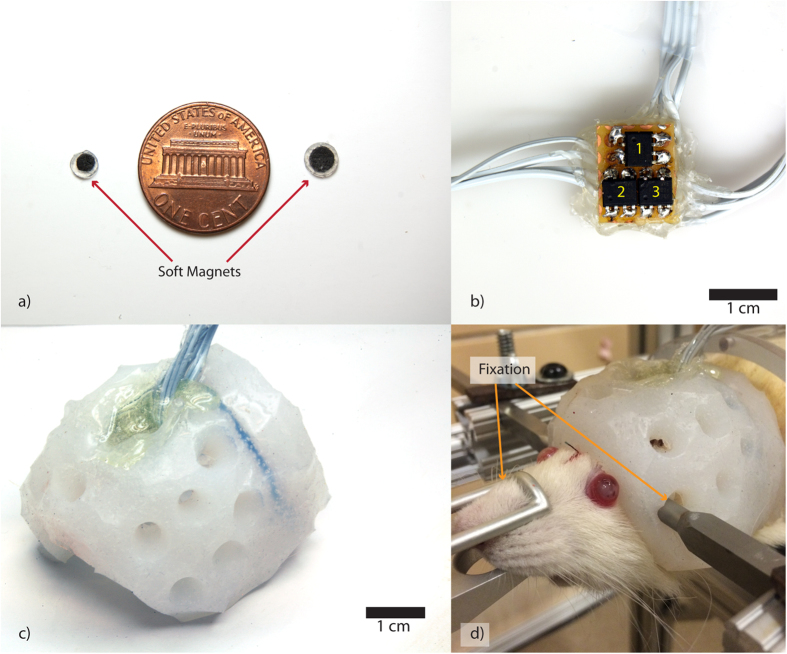
Intracranial brain deformation sensor system components. (**a**) Soft magnets of two different diameters (3 mm and 5 mm) (**b**). GMR array (from top going clock-wise are numbered from 1 to 3. (**c**) The polymeric helmet with embedded GMR sensors (**d**). An example of animal experiment setup. The rat’s head is fixed from the front and the sides. The helmet is strapped and later taped down.

The soft magnets were fabricated by loading ferromagnetic particles to a silicone elastomer and by magnetically polarizing the material during crosslinking (for details, see *Methods* and Supplementary Figure 1). The resulting magnets were 3 and 5 mm in diameters and 1 mm in thickness (Fig. 2a). The GMR array consists of three GMR sensors (labeled 1 to 3 going clockwise from the top) to allow three-dimensional tracking of the soft magnet (Fig. 2b). The overall array dimension is about 1 by 2 cm^2^ (see Supplementary Figure 2). The GMR array was then embedded into a helmet (Fig. 2c). The helmet was later used to fix the sensor array to the test subject, as shown in Fig. 2d.

### Soft Magnet Design

Because the system operates by tracking an implanted soft magnet, both magnetic and mechanical properties of the polymeric soft magnets are critical parameters. The soft magnet has to have sufficient magnetic strength to be measured wirelessly at reasonable distance. In addition, for the soft magnet to move with the brain during the deformation and not to injure the tissue, the Young’s modulus of the soft magnet needs to be similar to that of the brain (1–40 kPa depending on rate and magnitude of the impact)^13^,^15^. If too stiff, the soft magnet could penetrate the brain during deformation in a similar manner to a projectile, damaging the brain and compromising the accuracy and validity of the system. If too pliable, the soft magnet could be permanently deformed during bTBI after absorbing the impact during a blast event, which would potentially change the magnetic field in a manner inconsistent with our calibration efforts. (A detailed description of the characterization and the results are discussed in the supporting document). Following the characterization, it was shown that the soft magnets can retain the magnetic strength of 1 Gauss in PBS for four weeks and have Young’s modulus of 60 kPa (Supplementary Figure 3).

### Intracranial Brain Deformation Sensor System Calibration

**Figure 3:**
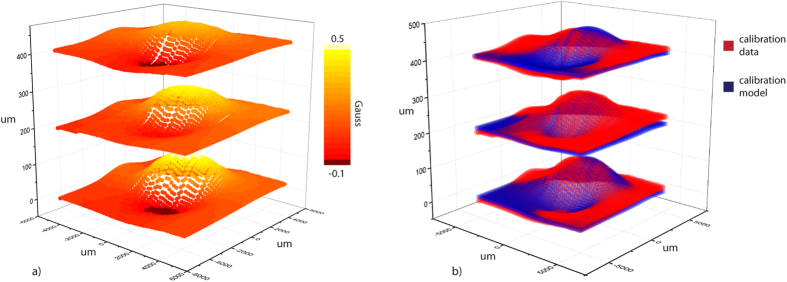
GMR sensor calibration. (**a**) Three horizontal slices of measured magnetic strength as a relative position of a soft magnet. (**b**) Calibration model using two 3-variable Gaussian obtained and the measured data are compared showing the validity of the calibration model.

The characterization of the GMR outputs as a function of the soft magnet positions was performed by scanning the soft magnet over the GMR sensor array with a motor-controlled micro-manipulator at a step size of 100 μm over a cubic space of 1 cm^3^. The voltage output of the GMR sensor was recorded as a function of the relative soft magnet position. Three slices of the scanned calibration map are shown in Fig. 3a. Each slice shows magnetic field strength measured by GMR sensor as a function of X-Y position at Z = 0, 200, and 400 μm. The color map indicates the magnetic field strength with respect to the Earth’s magnetic field. The calibration data was modeled as a sum of two 3-variable Gaussian, which accurately described the measurement as the two overlap in Fig. 3b. The equation and the parameters of the calibration model are provided in Supplementary Information. The position of the soft magnet, then, can be obtained by solving a solution satisfying Equation 1 (Supplementary Information) for a combination of three GMR outputs.

### Intracranial Brain Deformation Sensor System Validation

**Figure 4:**
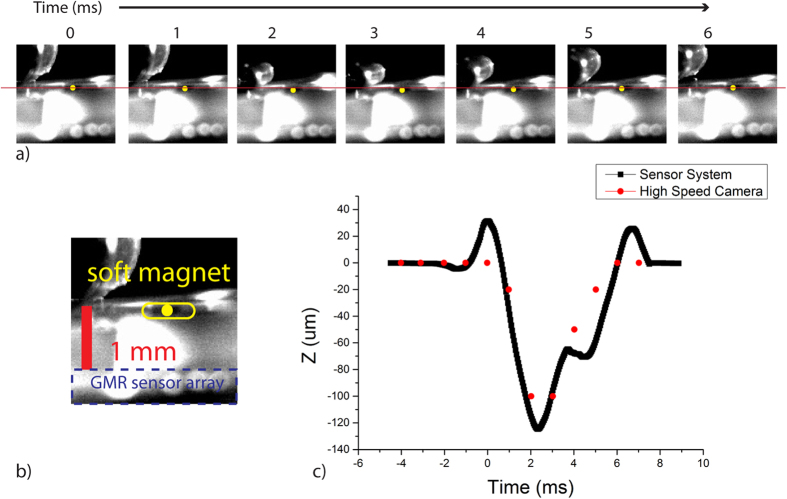
Validation experiment results. (**a**) Time evolution of the soft magnet displacement following the blast event. The yellow dots indicate the position of the soft magnet and the red line indicates the original position. Maximum deformation of around 100 μm between 2 and 3 ms was observed with a high speed camera. (**b**) Scale bar and the soft magnet location are shown. (**c**) Relative soft magnet positions measured with the sensor system and the high speed camera as a function of time.

To verify if the sensor system using the calibration model can accurately measure the position of a soft magnet, a quantitative validation experiment was performed. A soft magnet was embedded in a PDMS block of 1 mm thick sitting on top of the GMR sensor array and was exposed to a 150 kPa peak overpressure blast wave with 1.5 ms positive phase duration. The deformation of the PDMS block, and hence the movement of the embedded soft magnet, was monitored with a high speed camera. Simultaneously, the GMR sensor outputs were measured during the experiment. The validation experiment results are summarized in Fig. 4. The positions of the soft magnet for every one millisecond immediately following the blast are shown in Fig. 4a. The yellow dots and the red line indicate the soft magnets positions and the original position, respectively. It can be seen that the soft magnet moves down and returned to the original position. In Fig. 4b, the initial position of the soft magnet, the scale bar, and the GMR sensor array are shown. At 0 ms, the tape attached to the PDMS block moved, indicating that the blast wave had reached the tape, and the soft magnet was displaced as the PDMS block deformed. Maximum displacement of about 100 μm in vertical direction occurred between 2 and 3 ms (however, it was difficult to estimate the displacement of under 100 μm by pixel counting). Finally, within 6 milliseconds after the initial impact the soft magnet returned to its original position. The trajectory of the soft magnet measured by high speed camera is shown as the red dots on the Fig. 3c. The displacement of the soft magnet in vertical (z) direction (sampling rate of 25 kHz) obtained from the sensor system is plotted in the Fig. 4c in black solid line. At 0 ms and 6 ms, an upward peak in the GMR signal was observed, possibly a byproduct of the blast impact to the GMR sensor array. However, both the time scale and the displacement (120 μm) agree well with the images taken with the high speed camera validating the sensor system accuracy in measuring the soft magnet positions.

### Intracranial Brain Deformation Measurements with the Sensor System in Different Experimental Models

**Figure 5:**
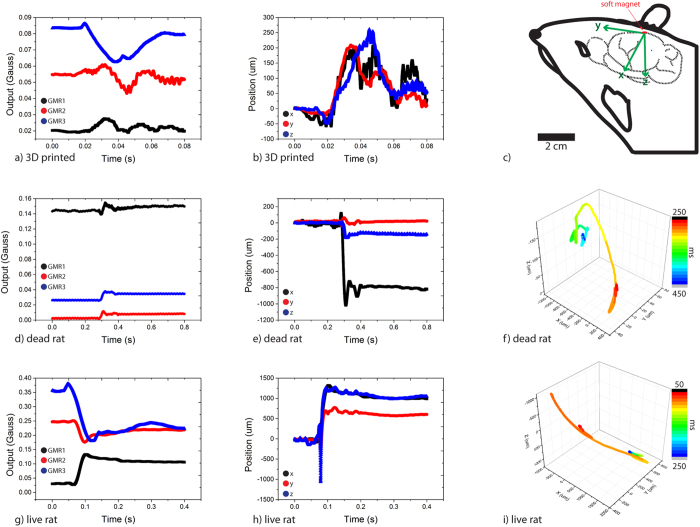
Summary of the sensor system measurements from three different test arrangements. (**a**) GMR sensor output. (**b**) Corresponding position of the soft magnet from 3D printed skull filled with agarose gel. (**c**) A representative figure showing the definition of coordinate axes with respect to the rat’s head. The dextrosinistral (ear-to-ear), anteroposterior (nose-to- tail), and dorsoventral (back-to-belly) axes were defined as x, y, and z, respectively. (**d**) GMR sensor output, (**e**) Corresponding position, and (**f**) Trajectory of the soft magnet under blast wave implanted in a dead rat are shown. (**g**) GMR sensor output, (**h**) Corresponding position, and (**i**) Trajectory of the soft magnet under blast wave implanted in a live rat are shown.

Following the sensor system validation experiment, the system was applied to different experimental models to measure intracranial brain deformations to study the primary bTBI. The experimental models were 3D printed rat skull filled with agarose gel, a dead rat, and a live rat. Each specimen was exposed to an incident blast overpressure wave with a maximum overpressure of 150 kPa and 1.5 ms positive phase duration delivered through an open-ended shock tube model. The GMR outputs, and the soft magnet position as functions of time obtained from the system, and the trajectory of the brain’s local deformation are plotted in Fig. 5 for all three arrangements. The obtained voltage outputs from each GMR sensor were converted to magnetic field by solving Equation 1 (Supplementary Information). The measured magnetic strength is converted to xyz position by calibration map with the initial position adjusted to zero. As a control experiment, the GMR sensor was exposed to blast overpressure wave mounted on the rat after taking the soft magnet out; in this case the GMR output was low, since there is no magnet. Even though there were some false signals generated from the blast impact, there was no appreciable difference in the magnetic field measured before and after the blast (Supplementary Figure 4). This indicates that the measured signals were generated from the experiment and the GMR sensor fixation was appropriate (since the change in the GMR orientation would cause the change in the measured Earth’s magnetic field).

The 3D printed rat skull filled with agarose gel (Supplementary Figure 5) was tested first. The signal from the GMR sensors and the corresponding position in 3-dimension are shown in Fig. 5a,b. Due to a long distance between the GMR array and the soft magnet, the signal appeared to be noisy since the voltage outputs from GMR sensors were low. Moreover, there was a second delayed peak, which is attributed to a delayed pressure wave reflected from top of the shock tube. The soft magnet exhibited sustained displacement of 50 μm within the time scale of 60 ms by the blast wave. For the animal experiments, a single coordinate system using of dextrosinistral (ear-to-ear; x-axis), anteroposterior (nose-to-tail; y-axis), and dorsoventral (back-to-belly; z-axis) axis was utilized. A representative coordinate axis with respect to the rat is shown in Fig. 5c.

Upon exposure to the blast wave, the dead rat’s brain showed a similar response, in nature, as the GMR outputs from each sensor are shown (Fig. 5d). The converted displacement shows the corresponding position of the soft magnet which provides the information regarding the local brain deformation at the implanted site (Fig. 5e). There was an arbitrary secondary peak observed likely due to the small electrical error (Fig. 5d), which is amplified due to conversion (Fig. 5e). This was because the sensor array was placed far from the soft magnet (~1 cm) making it susceptible to small electrical noise. The trajectory shows the initial deformation and the relaxation and a sustained deformation by a millimeter within 100 ms (Fig. 5f). The deformation was mostly in the dextrosinistral (ear-to-ear) direction accompanied by a small shift upward pressing into the skull. Lastly, a live rat was given a single soft magnet implantation and the position of the soft magnet was monitored utilizing the same experiment setup as the dead rat. The GMR outputs, corresponding soft magnet position, and the trajectory are summarized in Fig. 5g–i. The maximum deformation was mostly in the dextrosinistral direction, and in the dorsoventral direction. The sustained deformation of 1.6 mm occurred in the time scale of 100 ms.

## Discussions

The result indicates that a brain can be deformed by an exposure to blast wave even when the head and neck fixation prevent inertial acceleration effects, at least in rat models. Another interesting finding is that the displacements of the soft magnets were mostly in the dextrosinistral direction. It indicates that the deformations during bTBI may not be as simple as a blunt force trauma, but rather have a complex, multi-axial dynamic strain fields which have been shown to be more injurious to neurons than uniaxial deformation^16^,^17^. Compared to the dead rat’s brain, the brain of the live rat appeared to be more pliable and softer as the time scale of relaxation (about 100 ms compared to 90 ms) and the degree of sustained deformations (1.6 mm compared to about 1 mm) were both larger than those of the dead rat. It is expected, however, that on a longer time scale the living brain would completely relax back to its original position as fluid spaces re-equilibrate. These differences in the mechanical response, arising from the viscoelastic properties of the brain, under a blast wave could be attributed to the biological differences between the dead and live animals; even though the rats were sacrificed minutes before the implantation and subsequent experiments, the lack of perfusion and the drainage of the cerebral fluid could change the mechanical properties and cause stiffening of the brain^18^,^19^. The ability to capture post-mortem tissue stiffening behavior is another indication of the sensor’s capability of detecting the intracranial deformation of the brain during bTBI consistent with expected material behavior.

From these results, it is clear that the 3D printed skull-brain simulant, dead rat, and live rat exhibited a wide range of mechanical behavior. Table 1 provides a summary of major intracranial dynamics observations for each set of experiments. The observed results are consistent with expectations from existing literature. Brain tissue has a strain-rate dependent elastic modulus with more rigid behavior at higher strain-rates. Brain deformations during blast injury, though yet to be extensively studied, are expected to exhibit strain rates upward of 100/s^20^, levels which exceed reported damage thresholds at the cellular level^21^,^22^,^23^. Brain tissue viscoelastic behavior has been demonstrated to have moduli ranging from less than 1 kPa for quasistatic deformations (<0.1/s strain rate) up to 60 kPa for high rate dynamic deformations (3000/s strain rate)^20^, both of which are lower than 0.6% agarose at comparable strain rates^24^. The gel’s motion was additionally constrained due the fact that it completely filled its containing compartment, the 3D printed skull, whereas the brain in both the dead and live animals contained CSF fluid spaces around and within the brain which would allow for additional movement. All tissues, including the brain, have been consistently demonstrated to stiffen significantly post-mortem^18^,^19^. While the post-mortem time was minimized to the extent possible when testing the dead rat, this fact, paired with post-mortem lack of blood perfusion and consequential decreased water content, would be expected to raise the stiffness and solid-like characteristics of brain tissue behavior in the dead rat when compared to the live rat. Materials with lower stiffness and less solid-like behavior are less resistant to motion imposed by an outside force, thus reach higher internal deformations and velocities. By this logic, it is sensible that the greatest displacement and velocity were recorded in the live rat, while the smallest displacement and velocity were recorded in the 3D printed skull filled with agarose gel as reflected in Table 1.

However, due to the limited sensing distance (~1 cm), the GMR sensor array was exposed to the blast wave, causing an overestimation of the displacement at the moment of the impact. Although the effect was short-lived (<1 ms), this could be resolved by using different type polymer or different magnetic powder to increase the magnetic strength. Despite the limited sensing distance (~1 cm) , the system was successful in measuring the displacement of the soft magnet under blast wave, whose results reflected the difference in response due to the difference in mechanical properties of PDMS, agarose gel, and rat’s brain (validation, 3D printed, and rats).

This is the first report of direct *in situ* and *in vivo* monitoring of localized brain deformation under bTBI (at the implantation site) using animal models with a novel implantable sensor system. By implanting multiple soft magnets, the mapping of the brain deformation during bTBI could be obtained, although this would require further refinement of the method. However, the current size restriction imposed by rat anatomical features resulted in the present method to track only up to two soft magnets simultaneously. Moreover, in the present study, only surface soft magnet implantations (between dura and skull) were performed. This limitation was influenced by three major factors: magnetic field detection limits, vascular obstruction of subdural implantation, and potential for seizure on deep implantation. It is our current ongoing effort to address the limitations by increasing the magnetic strength of the soft magnet while reducing the form factor, refining the current surgical method, and identifying alternative methods to allow for deep implantation of the soft magnet into the brain.

Despite the abovementioned limitations, the presented system provides a unique tool in studying the brain’s mechanical behavior during bTBI. Understanding of brain’s mechanical deformation under bTBI is important, as it has been shown to damage neurons in both magnitude and rate-dependent fashions in other models of neural trauma. In the future, sub-dural implantations should be systematically explored to assess the mechanical response (stress, strain, strain rate, etc.) in a specific brain region of interest. Such efforts may identify brain regions predisposed to mechanical injury from primary blast exposure. Further, this system should be used to study traditional impact-acceleration TBI as well as combined primary and secondary bTBI to directly compare the brain’s mechanical behavior between different traumatic brain injury modalities. This novel sensor system will allow for testing hypotheses regarding pathogenesis post-TBI neuropathologies that have been largely speculative and shed light on potential methods for injury prevention, diagnosis, and treatment.

## Methods

All animal procedures were carried out in accordance with animal experimental protocols approved by the Purdue Animal Care and Use Committee (Protocol #1111000280).

### Soft magnet preparation

The soft magnets were fabricated by mixing silicone elastomer with iron oxide (Fe_2_O_3_) nanoparticles and a subsequent magnetization during the polymerization. Four different particle concentrations (weight percentage) were tested; 20, 30, 40 and 50%. Generally, the soft magnets became magnetically stronger and mechanically stiffer as the concentration increased. Two loading concentrations, 30 and 40%, provided a good combination of mechanical and magnetic properties; whereas the 20% and 50% were too magnetically weak or mechanically brittle, respectively. The fabrication process is illustrated in Supplementary Figure 1. Commercially available silicone elastomer (Ecoflex, Smooth-On) was mixed with iron oxide nanoparticles (Sigma Aldrich) and the mixture was poured into an acrylic mold. A pair of disk magnets (3 mm in diameter) were placed on the top and the bottom of the mold to align the nanoparticles to magnetize the polymer mixture. Following magnetization and polymerization, the permanently magnetized film was removed from the mold, and was coated with a thin layer of silicone elastomer to form the soft magnets. The last layer of the coating was added so that the soft magnets can remain stable in the biological environments.

### GMR sensor array

Three GMR sensors (AAH002-02E, NVE Corp.) were arranged in a triangular configuration to track the movement of the soft magnets implanted on dura mater. The configuration of the sensor arrangement is illustrated in Supplementary Figure 2. The sensor dimension is 5 mm by 3.8 mm with a height of 1 mm. Two sensors are aligned laterally along the x-axis (blue line) with 0.2 mm of spacing, and the third sensor is spaced out from the midpoint of the two sensors along the y-axis by 1.8 mm of spacing. Utilization of three GMR sensors allows for measurement of displacement in three dimensions. For instance, when the soft magnet moves away from the sensor array, in z-direction in Supplementary Figure 2, the outputs of all three sensors decrease. One the other hand, displacements in both x and y directions can be tracked by looking at the ratio of the outputs of the GMR sensors. In the experiment with dead and live rats, the GMR sensor array was embedded to a porous, polymeric helmet to fix the sensor array to the skull.

### GMR array calibration

A soft magnet of diameter of 3 mm was placed on a GMR sensor horizontally, and the distance between the two was modulated using a motor controlled manipulator. A volume of 1 cm^3^ above the center of the GMR sensor was scanned with a 100 μm step size, while the GMR output was simultaneously measured at each point.

### Brain deformation calculation

The three GMR sensors provide a three dimensional reading of the soft magnet position. Each GMR sensor, based on the measured magnetic field strength, can provide potential positions of the soft magnet based on the calibration model, illustrated in Fig. 3 and written in Equation 1 (Supplementary). The soft magnet position can be calculated by solving a series of equations simultaneously, Equation 2 in Supplementary Information. From the calculated position of the soft magnet, the deformation of the brain (corresponding to the displacement of the soft magnet) can be obtained. All position results presented in this paper (Figs 4 and 5) were obtained using this calculation method.

### Blast wave overpressure generation

Blast waves were generated using a validated open-ended shock tube model by compressing nitrogen into a chamber with one end sealed by a thin (0.25″) PET membrane^14^. When the pressure inside the chamber exceeds the mechanical strength of the membrane, the membrane breaks. As a result, the compressed nitrogen is released through the shock tube generating a shock wave that exhibits characteristics mimicking the ideal Friedlander waveform used to describe shock wave phenomena^14^,^25^. While a single shock wave was chosen for this study consistent with blast injury models from other investigations and expert recommendations^26^, altering the thickness of the membrane as well as chamber sizes would allow for tunable shock wave parameters such as overpressure magnitude and positive phase duration^27^.

### Validation Experiment

The displacement measured with the sensor system was compared to the images taken with the high speed camera (Vision Research Phantom Camera v7.1M, 66666 frames per second). A tape was attached to the top of the PDMS block to optically indicate the time when the blast wave hit the sample. For this validation experiment, only the vertical displacement was considered since displacements in other directions were difficult to quantify with a single high speed camera.

### Surgical Opening (Craniotomy)

Rats were placed in a prone position and the head was shaved prior to fixation in a stereotaxic frame (Kopf Instruments). A midline rostro-caudal skin incision was made using a scalpel (11 blade) from the interorbital skin rostrally to the base of the skull caudally. The scalpel was used to dissect the underlying connective tissue and curved hemostatic clamps were used to retract the skin edges in order to achieve adequate exposure of the skull. The surface of the skull was cleaned by removing any additional connective tissue via blunt dissection and wiping with a gauze sponge. Bregma, lambda, and the temporalis muscles were identified before proceeding.

A handheld dremel with a dental burr (carbide bur 557, Henry Schein) was used to make a shallow outline of an oval area for craniotomy which extended from lambda to bregma and bilaterally to the edges of the temporalis muscles. Using this outline as a guide, the burr was used to cut a skull flap which was then carefully removed using a spatula and forceps (Supplementary Figure 6). After removal of the skull flap, the dura mater was identified and verified to be intact. Hemostasis was achieved using direct pressure with sterile gauze if needed. For implantations superficial to the dura as presented in this manuscript, no further opening steps were required. The sensor was then placed at the desired location on the brain surface.

### Surgical Closing (Cranioplasty)

The skull flap that was removed during the opening procedure was placed back in its original position. A small amount (variable between subjects) of acrylic bone cement powder (Simplex P, Stryker Instruments) was carefully placed into the gap between the replaced skull flap and the remainder of the skull. Then, the hardening solution was applied into the powder-filled gap via dropper pipet at a 1:2 ratio with the powder and allowed to cure for ten minutes. After ten minutes, cranioplasty integrity was assessed by pressing with forceps at gradually increasing levels of force until resistance to firm pressure was confirmed in the center and at the periphery of the flap.

### Animal Experiment Setup

Following the implantation surgeries, the GMR sensor array was fixed to the skull using a helmet and the wires were taped down. In the experiment with a live rat, the rat was anesthetized prior to the implantation and exposures to blast waves. After the experiment, the rat was sacrificed. The rats were held down by an acrylic body holder serving as both a protective gear and an immobilizer, and their heads were immobilized using a stereotaxic head fixation to isolate primary shock wave-induced deformation from blast wind acceleration-induced brain motion. The blast tube was placed right above the head within one shock tube diameter of the outlet for optimal shock wave conditions^28^. Lastly, the GMR array embedded helmet was strapped onto the rats. After sacrificing the rat following the blast experiment, the top of the skull was removed to ensure the implanted soft magnet did not penetrate through dural or brain tissue and to assess any visible deformation to the brain. Although signs of deformation due to the blast were evident as indicated by the measurements, the soft magnet remained superficial to the dura at the implantation site and did not penetrate the tissue, confirming the sensor moved *with* and not through the tissue during bTBI.

## Additional Information

**How to cite this article**: Song, S. *et al.* A Wireless Intracranial Brain Deformation Sensing System for Blast-Induced Traumatic Brain Injury. *Sci. Rep.* **5**, 16959; doi: 10.1038/srep16959 (2015).

## Supplementary Material

Supplementary Information

## Figures and Tables

**Table 1:** Summary of intracranial dynamics during bTBI experiments.

Model	Max Displacement	Max Velocity	Sustained Displacement
*3-D Printed Skull*	350 μm (at 20 ms)	23 mm/s	50 μm (at 60 ms)
*Dead Rat*	1.2 mm (at 25 ms)	50 mm/s	1 mm (at 90 ms)
*Live Rat*	2.1 mm (at 30 ms)	70 mm/s	1.6 mm (at 100 ms)
