# Supplementary material for: A Wireless Intracranial Brain Deformation Sensing System for Blast-Induced Traumatic Brain Injury
Source: Sci Rep. 2015 Nov 20;5:16959. doi: 10.1038/srep16959 (PMC4653713; doi:10.1038/srep16959)
Supplement: Supplementary Information [file srep16959-s1.doc]

**Supplementary Information**

A Wireless Intracranial Brain Deformation Sensing System for Blast-Induced Traumatic Brain Injury

S. Song^1, 2†^, N. S. Race^3, 5†^, A. Kim^1, 2†^, T. Zhang^1, 2^, R. Shi^3, 4*^, and B. Ziaie^1, 2*^

^1^School of Electrical and Computer Engineering, Purdue University, West Lafayette, IN, USA

^2^Birck Nanotechnology Center, West Lafayette, IN, USA

^3^Weldon School of Biomedical Engineering, ^4^Department of Basic Medical Sciences, Purdue University, West Lafayette, IN, USA

^5^School of Medicine, Indiana University, Indianapolis, IN, USA

Authors denoted by ^*^ Co-correspondent authors

Authors denoted by † contributed equally to the work.

Soft magnet preparation

Soft magnets were prepared in two different loading concentrations (30 and 40%) in a rectangular film of 5 X 5 X 0.8 mm^3^ for strain-stress characterization. The strains of the soft magnet films were measured under different static loads using a dynamic mechanical analysis system (Q800, TA Instruments, USA). Figure 6 shows the strain vs. stress curves for the soft magnets of two different loading concentrations. It was observed that with a higher loading concentration, the material became stiffer and more brittle as evident in the differences in the slopes at the linear regions and the yielding strains. The elastic moduli were measured to be 60 and 80 kPa for 30 and 40 particle percentages, respectively. These moduli are higher than the 1 – 40 kPa range for brain tissue (strain rate dependent), but they are comparable to the upper end of high strain-rate brain tissue elastic moduli (40 kPa) and orders of magnitude lower than conventional metal-fabricated sensors.


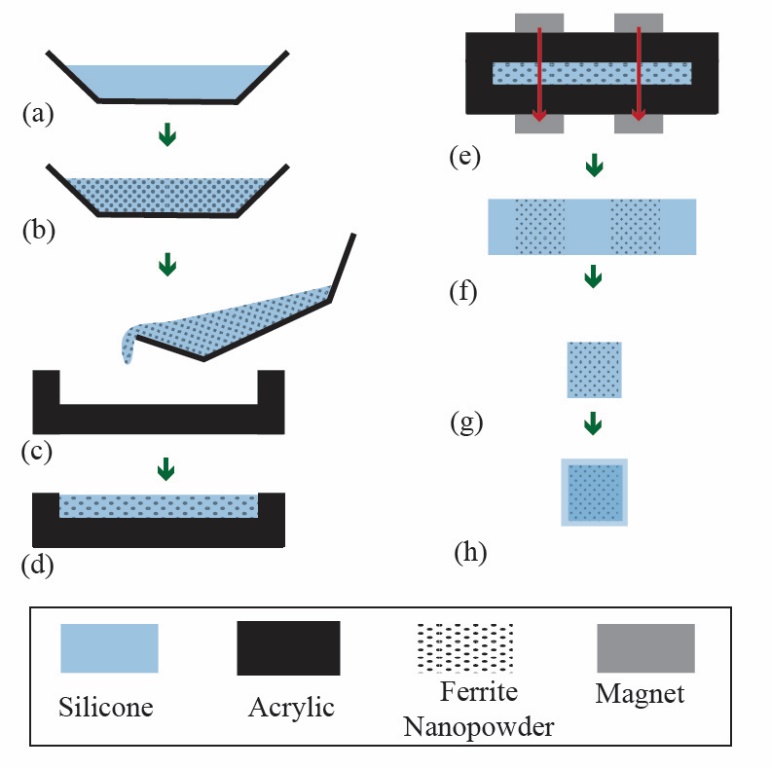


Supplementary Figure 1. Soft Magnet fabrication process. (a) silicone elastomer is prepared; (b) iron oxide nanoparticles are added; (c) acrylic mold is prepared; (d) the mixture is poured into the mold; (e) magnetization during the polymerization; (f) removal from the mold; (g) the magnetized film is cut into desired size; (h) additional coating of silicone is applied.

GMR Array Configuration


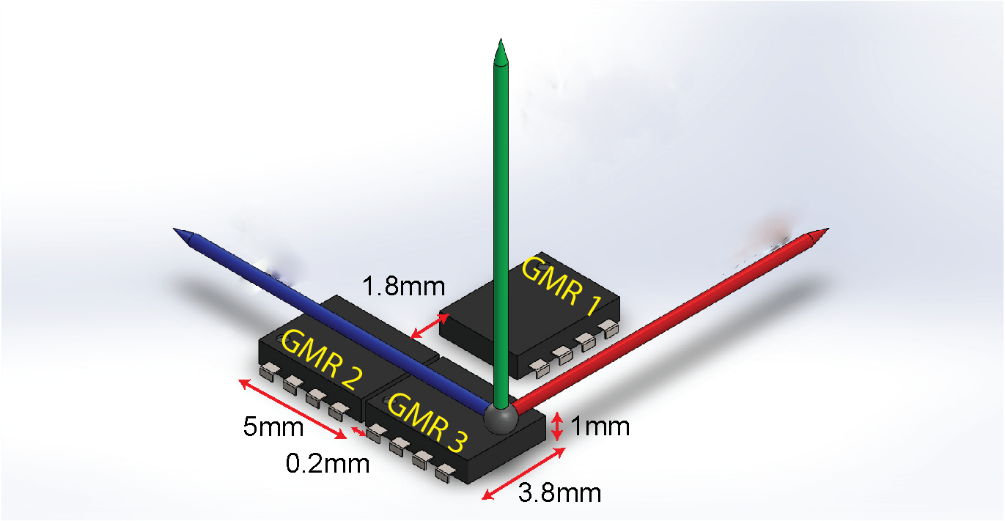


Supplementary Figure 2. GMR Sensor Array Configuration

Soft Magnet Characterization

To verify the stability of the soft magnet, the magnets were kept in the PBS (phosphate buffered saline) solution kept at 37 ℃ and their magnetic strengths were measured daily. After going through the initial decay, the strength of the soft magnets stabilized around 0.7 – 0.9 gauss (Supplementary Figure 3). However, sample to sample variations were quite large because controlling the aggregation of the iron oxide nanoparticles under the polling magnetic field was difficult. After the characterization, 30% soft magnets were chosen to be utilized in the intracranial brain deformation sensor system for its excellent magnetic and mechanical properties.


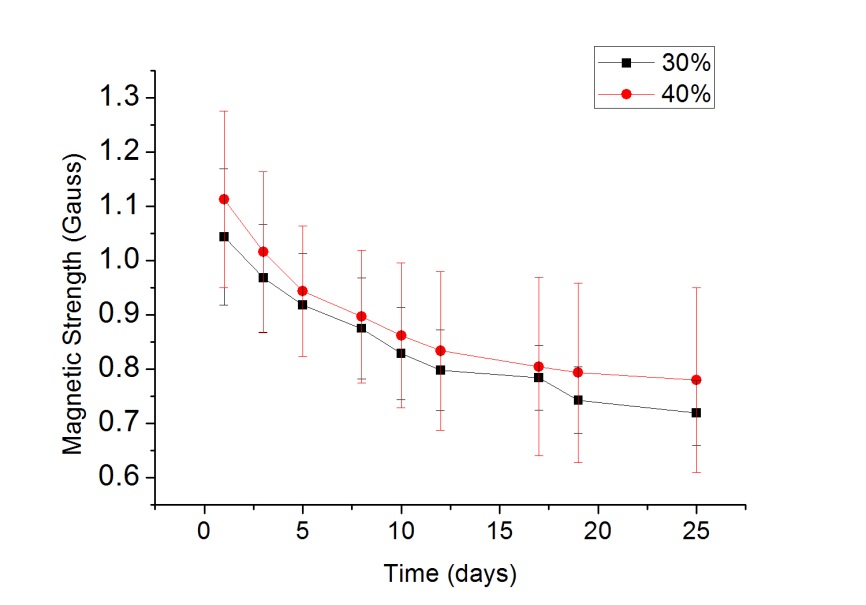

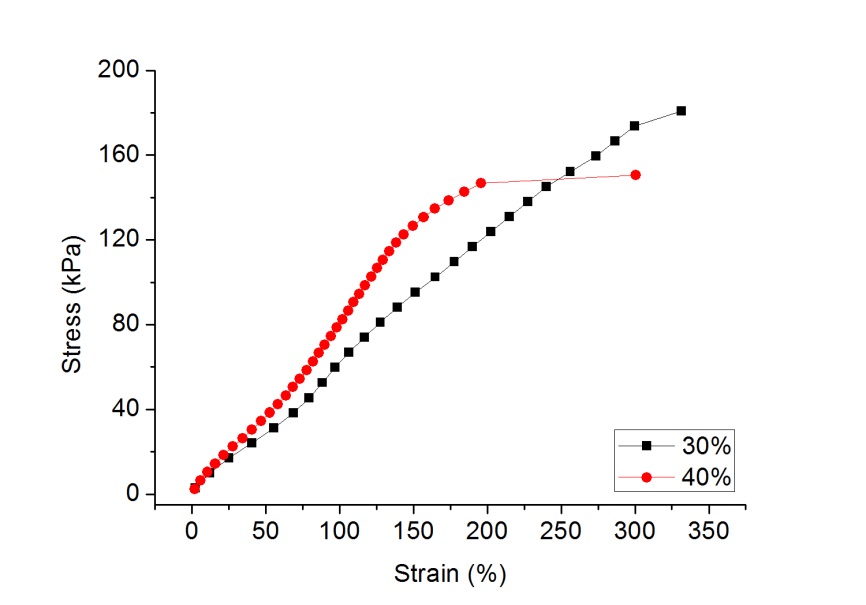


Supplementary Figure 3. a) soft magnet stability test in PBS; b) strain vs. stress plot of soft magnets with two different loading concentrations

Equation 1. Magnetic field strength as a sum of two 3-variable Gaussian distribution


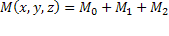


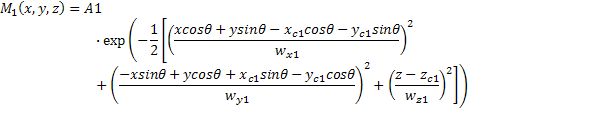


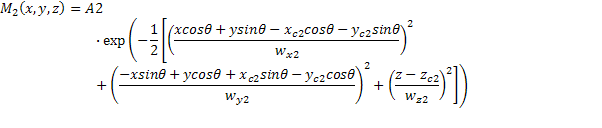


Table 1. Gaussian Parameters

| M0 | 0.059 |  | |
| --- | --- | --- | --- |
| A1 | 2.8 | A2 | -0.95 |
| Xc1 | 200 | Xc2 | 500 |
| Yc1 | 1300 | Yc2 | -1630 |
| Zc1 | -3817 | Zc2 | -3817 |
| Wx1 | 2000 | Wx2 | 1600 |
| Wy1 | 1000 | Wy2 | 2000 |
| Wz1 | 2090 | Wz2 | 2090 |
| theta1 | 0.5 | theta2 | 0 |

From each GMR output, a set of possible positions of the soft magnet can be inferred by solving Equation 1. However, to narrow down the set of solutions to a single point, a series of equations can be solved simultaneously for all three GMR sensors to obtain the soft magnet solution.

Equation 2. A series equation for obtaining soft magnet position in three dimension.


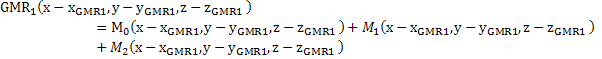


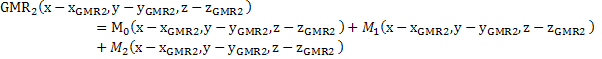


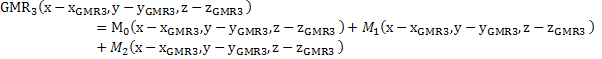


By solving the three equations simultaneously, the position (x, y, z) can be obtained, where (x_GMRn_, y_GMRn_, z_GMRn_) denotes the relative position of the GMR sensor in respect to the reference point.


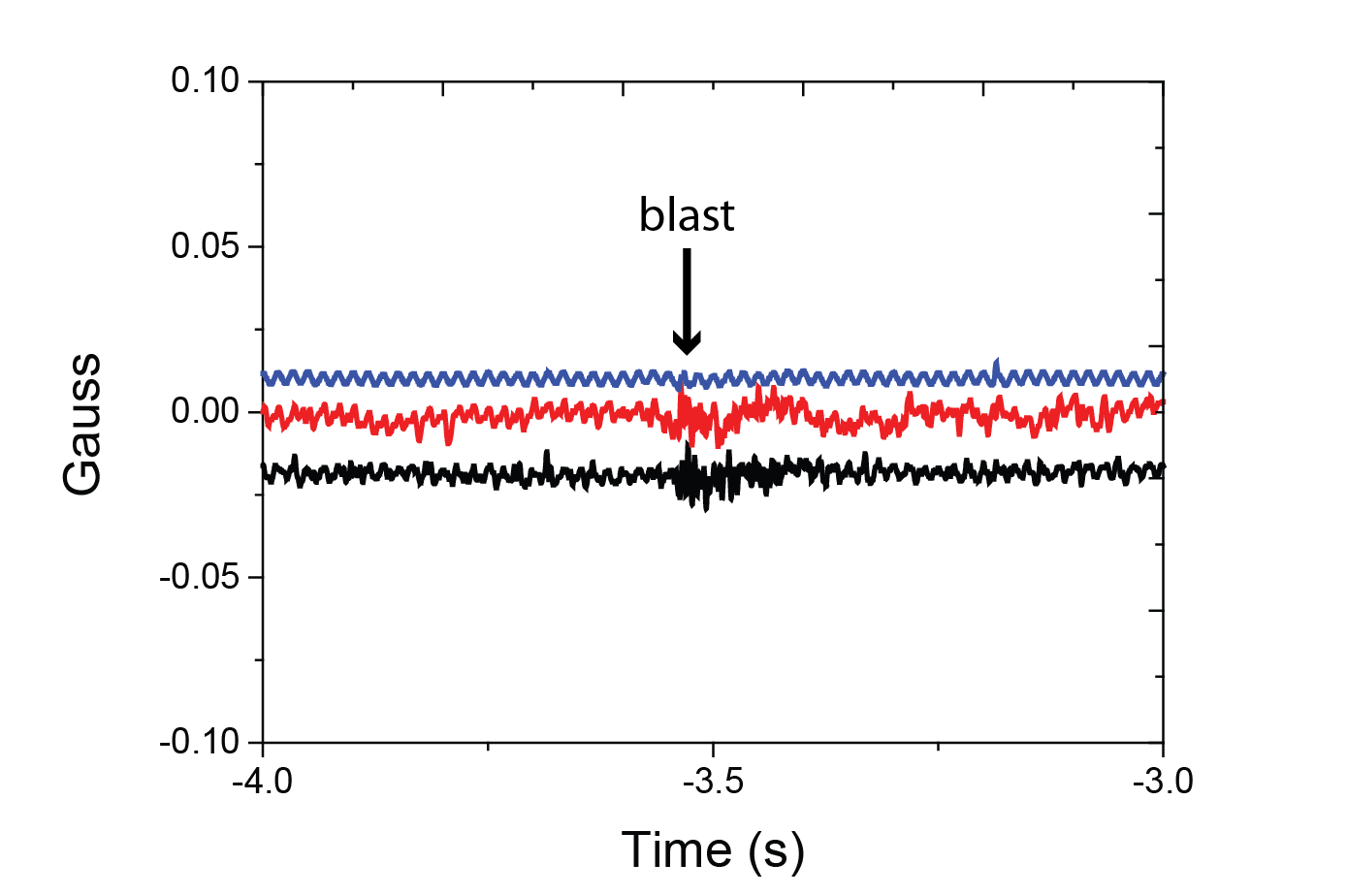


Supplementray Figure 4. Control experiment. Following the dead rat experiment, the soft magnet was taken out. The same experiment setup was utilized with the rat fixed under the blast system with the GMR sensor array fixed to the skull. The array indicates the time that the blast wave was delivered. There was no appreciable change due to the blast except the electrical noise.


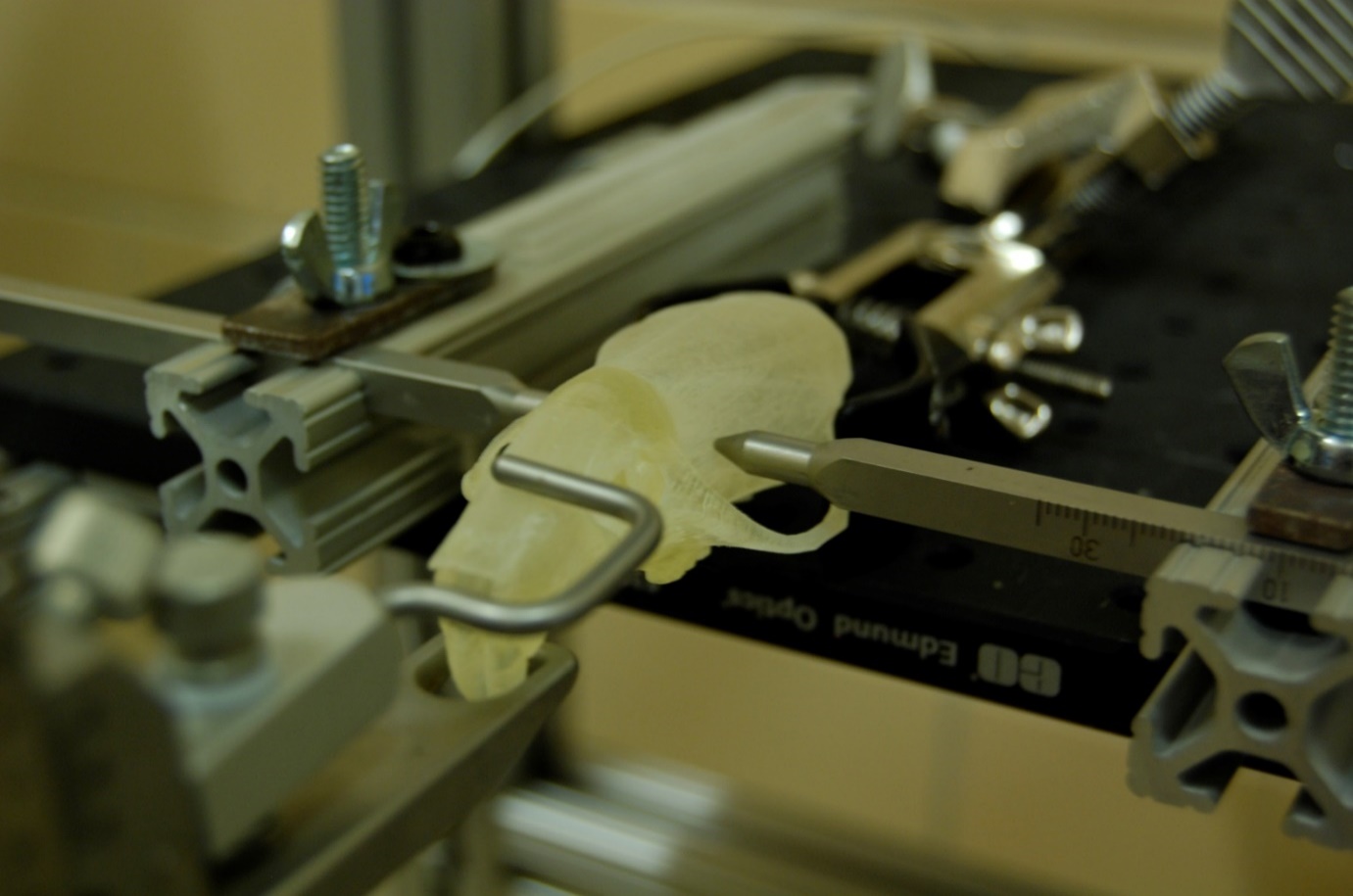


Supplementary Figure 5. Optical picture of 3D printed skull filled with agarose gel fixed under blast nozzle.


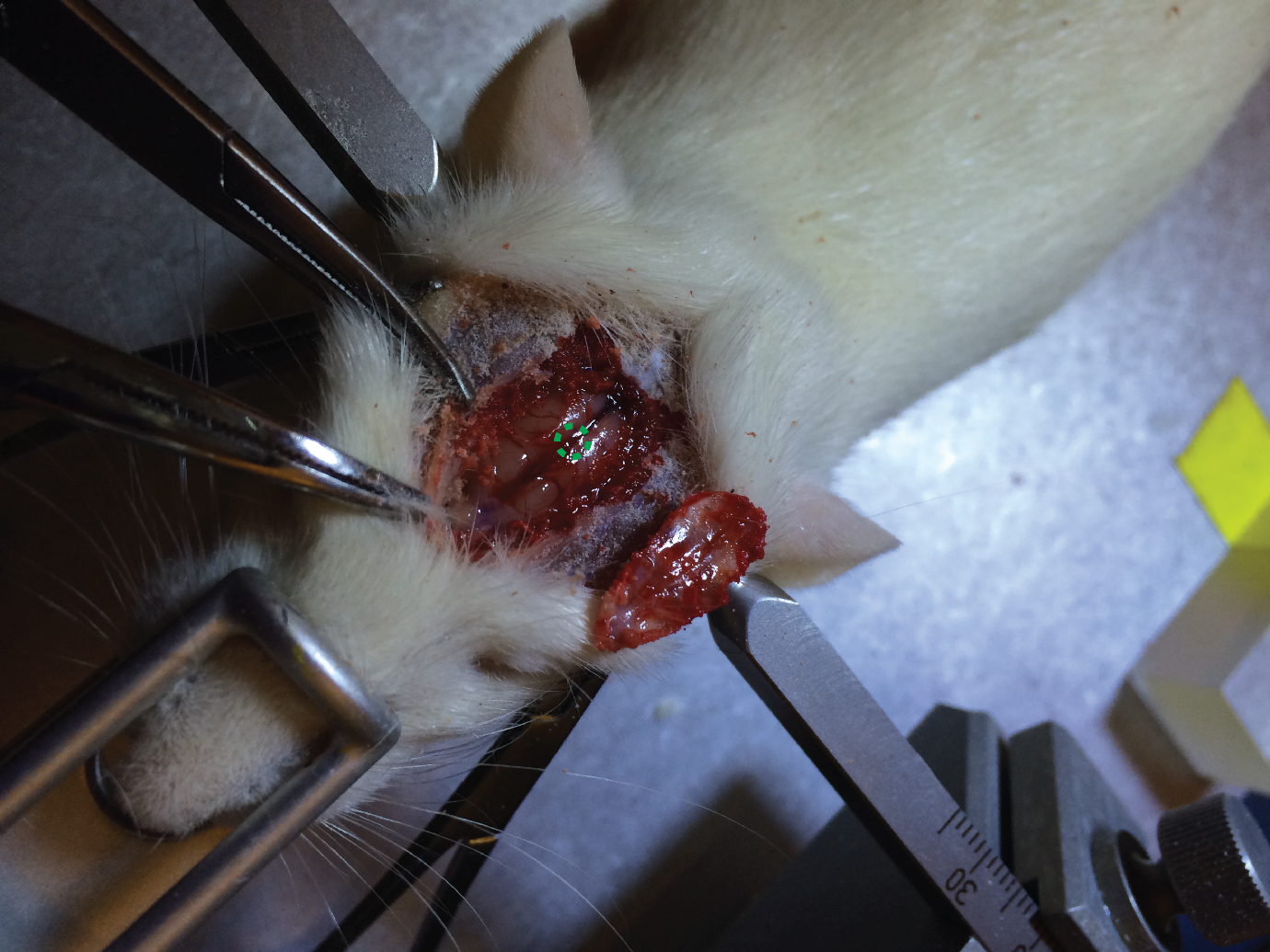


Supplementary Figure 6. Picture taken during implantation of soft magnet on to the dura mater. The rat was sacrificed just before the surgery. The typical implantation site is outlined by a circle.
